# Identifying Candidate Genes Involved in the Regulation of Early Growth Using Full-Length Transcriptome and RNA-Seq Analyses of Frontal and Parietal Bones and Vertebral Bones in Bighead Carp (*Hypophthalmichthys nobilis*)

**DOI:** 10.3389/fgene.2020.603454

**Published:** 2021-01-15

**Authors:** Weiwei Luo, Ying Zhou, Junru Wang, Xiaomu Yu, Jingou Tong

**Affiliations:** ^1^State Key Laboratory of Freshwater Ecology and Biotechnology, Institute of Hydrobiology, The Innovation Academy of Seed Design, Chinese Academy of Sciences, Wuhan, China; ^2^College of Advanced Agricultural Sciences, University of Chinese Academy of Sciences, Beijing, China

**Keywords:** bighead carp, differential growth, Iso-Seq, full-length transcript, differentially expressed unigene

## Abstract

Growth, one of the most important traits monitored in domestic animals, is essentially associated with bone development. To date, no large-scale transcriptome studies investigating bone development in bighead carp have been reported. In this study, we applied Isoform-sequencing technology to uncover the entire transcriptomic landscape of the bighead carp (*Hypophthalmichthys nobilis*) in early growth stage, and obtained 63,873 non-redundant transcripts, 20,907 long non-coding RNAs, and 1,579 transcription factors. A total of 381 alternative splicing events were seen in the frontal and parietal bones with another 784 events simultaneously observed in the vertebral bones. Coupling this to RNA sequencing (RNA-seq) data, we identified 27 differentially expressed unigenes (DEGs) in the frontal and parietal bones and 45 DEGs in the vertebral bones in the fast-growing group of fish, when compared to the slow-growing group of fish. Finally, 15 key pathways and 20 key DEGs were identified and found to be involved in regulation of early growth such as energy metabolism, immune function, and cytoskeleton function and important cellular pathways such as the arginine and proline metabolic pathway (*p4ha1*), FoxO signaling pathway (*sgk1*), cell adhesion molecules (*b2m*, *ptprc*, and *mhcII*), and peroxisome proliferator-activated receptor signaling pathway (*scd*). We established a novel full-length transcriptome resource and combined it with RNA-seq to elucidate the mechanism of genetic regulation of differential growth in bighead carp. The key DEGs identified in this study could fuel further studies investigating associations between growth and bone development and serve as a source of potential candidate genes for marker-assisted breeding programs.

## Introduction

Growth is one of the most economically important traits monitored in domestic animals and therefore is the main objective of most genetic selection programs. Growth rates of aquatic animals, as well as of most organisms, are regulated by multiple genetic and environmental factors (Cleveland et al., 2020). Over the past few decades, the genetic basis of growth in aquatic species has been widely studied. Genetic linkage analyses for genes regulating growth in aquatic animals have been performed using DNA-based molecular markers such as amplified fragment length polymorphism and simple sequence repeat (SSR) (Liu and Cordes, 2004; Yue, 2014). With the development of next-generation sequencing technology, quantitative trait loci (QTL) mapping and genome-wide association study have allowed for the identification of genetic markers associated with the traits of interest. Additionally, transcriptome analyses can be used to identify biomarkers and candidate genes regulating growth. A total of 92 differentially expressed genes (DEGs) were identified between fast-growth and slow-growth families of blunt snout bream (*Megalobrama amblycephala*) in transcriptomes of the liver and gill (Li et al., 2015). Cleveland et al. (2020) compared liver and muscle transcriptomes of a rainbow trout (*Oncorhynchus mykiss*) line, selectively bred for fast growth, to that of a contemporary randomly mated control line and identified 145 and 36 DEGs in the liver and white muscle transcriptomes, respectively. Our laboratory has also identified 173 DEGs in the hypothalamus–pituitary and 204 DEGs in the liver of bighead carp with differential growth rates (Fu B. et al., 2019). However, these studies have mainly focused on gene expression patterns in the muscle, liver, and brain tissues. Growth, however, is essentially associated with skeleton and bone development and leads to consequent increases in body weight (BW) and body length (BL). The frontal and parietal bones are important components of the skull in vertebrates, serving to protect the brain and permit skull expansion during development (Quarto et al., 2009; Topczewska et al., 2016). The presence of vertebral bone discriminates vertebrate organisms from invertebrates. It supports the BW of the animals and generates force in the muscles for locomotion and physiological activities (Ibaraki et al., 2015). To our knowledge, no large-scale transcriptomic study investigating the differential expression of genes, regulating differential growth rates, in the bones of bighead carp has been previously reported.

Bighead carp (*Hypophthalmichthys nobilis*), one of the most important fish in Asia, improves the water quality for human consumption and thus has been introduced in many non-Asian countries (Szabo et al., 2019). Principally produced in China, the global production of bighead carp reached 3.1 million tons in 2017 (FAO). The size of the head in aquaculture fish species is important not only for understanding evolution and biological adaptation but also for predicting the economic value, as it directly affects filet yield (Geng et al., 2016). For most aquaculture fish species, smaller head and uniform body conformations provide a greater proportion of carcass yield; thus, selection for smaller head and streamline body conformation is of great value in aquaculture (Rutten et al., 2005). However, the bigger head of bighead carp harvest fish is more popular among Chinese consumers and is used as a source of nutrition in soups (fish head and tofu soup) or delicious dishes (steamed fish head with diced hot red peppers and so on). Therefore, to meet the demands of markets and farmers, selection for fast-growing and big-headed varieties of bighead carp is prevalent among Chinese breeding programs.

Transcriptomic analyses, widely used in genetic research, uncover the type and expression levels of transcripts and identify physiological and biochemical processes that regulate transcription (Jia et al., 2018). However, owing to technical limitation, obtaining the full-length transcripts and quantifying the isoforms are not possible with RNA sequencing (RNA-seq) (Steijger et al., 2013). Thus, single-molecule real-time (SMRT) sequencing (Pacific Biosciences of California, Inc., Menlo Park, CA, United States) has been developed (Korlach et al., 2010), and it provides a third-generation sequencing platform that is widely used to sequence genomes by generating kilobase-sized sequencing reads (Chaisson et al., 2015; Gong et al., 2018). Moreover, recent studies have reduced the high error rate (up to 15%) of SMRT sequencing by self-correction, via circular-consensus sequencing reads (Li et al., 2014) and validation with high-throughput and high-accuracy short-read data (Au et al., 2013). SMRT sequencing comprehensively analyses splice isoforms of each gene and improves the annotation of existing model organisms by producing full-length transcripts (Tilgner et al., 2014; Wang et al., 2016; Mehjabin et al., 2019). In this study, we applied SMRT sequencing to uncover post-transcriptional regulatory events in bighead carp and coupled it with bone RNA-seq to investigate the genetic regulation of early growth at the transcript level. This is the first study to report the application of SMRT sequencing in bone tissues and provides a comprehensive view of transcriptome complexity in bighead carp at the early growth stage. Furthermore, we identified a set of differentially expressed unigenes (DEGs) from bone development and body growth-related signaling pathways, which potentially services as valuable resources for future molecular breeding studies.

## Results

### Morphological Measurement

In total, six bighead carp individuals in juvenile stage, including three fast-growing [big group, (BG)] and three slow-growing [small group (SG)] fish, were selected for transcriptome analysis in this study. The BL, head length (HL), head height (HH), head width (HW), and BW were measured (Table 1). The ratios of HL and BL (HL/BL) from the BG and SG were 0.3239 and 0.3533, respectively. The average BLs for BG and SG were 21.30 and 16.13 cm, respectively. BL from BG was significantly larger than that from SG (*p* < 0.05). Other phenotypic parameters from BG were significantly larger than those from SG, including HL, HH, HW, and BW (*p* < 0.05).

**TABLE 1:** Phenotype differences of bighead carp between big and small groups.

**Character**	**BL (cm)**	**HL (cm)**	**HH (cm)**	**HW (cm)**	**BW (g)**
BG	21.30 ± 0.20*	6.90 ± 0.10*	6.10 ± 0.15*	4.23 ± 0.06*	185.90 ± 5.15*
SG	16.13 ± 0.06	5.66 ± 0.15	4.80 ± 0.05	3.26 ± 0.04	80.97 ± 0.45

BG is the abbreviation of big bighead carp and SG means small bighead carp. *Significant difference (*p* < 0.05) between BG and SG. BL, body length; HL, head length; HH, head height; HW, head width; BW, body weight.

### Full-Length Transcripts From the Bone Tissues

Two full-length transcriptomes of bighead carp with different growth rates were generated using the PacBio Sequel platform on the pooled RNA from each of three frontal and parietal bones and vertebrae in BG and SG, respectively. F01 represented the full-length transcriptome from each of three frontal and parietal bones and vertebrae in BG; F02 represented the full-length transcriptome from each of three frontal and parietal bones and vertebrae in SG. A total of 27.06 and 30.11 G subread bases were generated by two SMRT cells from the PacBio library of BG and SG, respectively. Under the conditions of full passes of ≥3 and quality of >0.9, 374,738, and 402,340 circular consensus sequence (CCS) reads were identified in F01 and F02, with the mean read lengths of 1,521 and 1,772 bp, respectively. CCS reads of F01 and F02 comprised 225,336 full-length non-chimeric (FLNC) reads and 295,486 FLNC reads, respectively. Based on the iterative clustering for error correction (ICE) Quiver and arrow polishing algorithms, we produced 72,625 and 93,838 polished full-length consensus transcripts from F01 and F02 with the mean lengths of 1,496 and 1,741 bp, respectively. Percentages of polished high-quality isoforms from F01 and F02 were 96.13 and 96.50%, respectively. After correction using short reads produced by Illumina (San Diego, CA, United States), short-read RNA-seq, and subsequently removing redundancies using the CD-Hit program, the consensus transcripts were finally clustered into a total of 63,873 non-redundant transcripts for subsequent analysis, including 36,584 and 44,852 non-redundant transcripts from F01 and F02, respectively.

### Sequencing and Quality Assessment of Short Reads

Twelve Illumina RNA-seq libraries constructed from frontal and parietal bones and vertebrae of bighead carp with different growth rates (BG and SG) were sequenced to correct the polished CCS and to quantify full-length transcripts obtained from PacBio Iso-Seq. After trimming process and screening with the high-quality reads, a total of 92.04 G clean reads were produced from all samples. An average of 80.67, 61.25, 78.78, and 86.66 million clean reads were obtained from the frontal and parietal bone cDNA libraries of BG (BF group), frontal and parietal bone cDNA libraries of SG (SF group), vertebra cDNA libraries of BG (BV group), and vertebra cDNA libraries of SG (SV group), respectively. In total, 24.16, 18.33, 23.60, and 25.94 Gb of clean bases were generated in the BF, SF, BV, and SV groups, respectively. The Q30 value of each sample was up to 93.03%, and the GC distribution of each sample ranged from 47.22 to 50.64% (Supplementary Table 1). The results showed that the sequencing quality was high, and the data could be used for subsequent analyses.

### Efficient Gene Annotation of Full-Length Transcripts

To obtain a comprehensive functional annotation from the full-length bone transcriptome of bighead carp, all the 63,873 non-redundant transcripts were assigned to align with eight different databases, including NCBI non-redundant protein sequences (NR), euKaryotic orthologous groups (KOG), Cluster of Orthologous Groups of proteins (COG), Protein family (Pfam), Swiss-Prot (a manually annotated and reviewed protein sequence database), the Kyoto Encyclopedia of Genes and Genomes (KEGG) orthology, Gene Ontology (GO), and eggNOG. A total of 68.49% of the non-redundant transcripts (43,747 of 63,873 transcripts) were successfully annotated with significant hits (*E* value < 1*E*^–5^) from these databases. The statistics of the full-length transcript annotations are listed in Table 2. The remaining unannotated transcripts (20,126 transcripts) might represent novel bighead carp species-specific genes.

**TABLE 2:** Summary of annotations on non-redundant transcripts from bone tissues against public databases.

**Annotated database**	**Annotated number**
COG annotation	11,755
GO annotation	34,791
KEGG annotation	25,955
KOG annotation	30,394
Pfam annotation	35,229
Swiss-Prot annotation	28,351
eggNOG annotation	41,726
Nr annotation	43,527
All annotated	43,747

**FIGURE 1:**
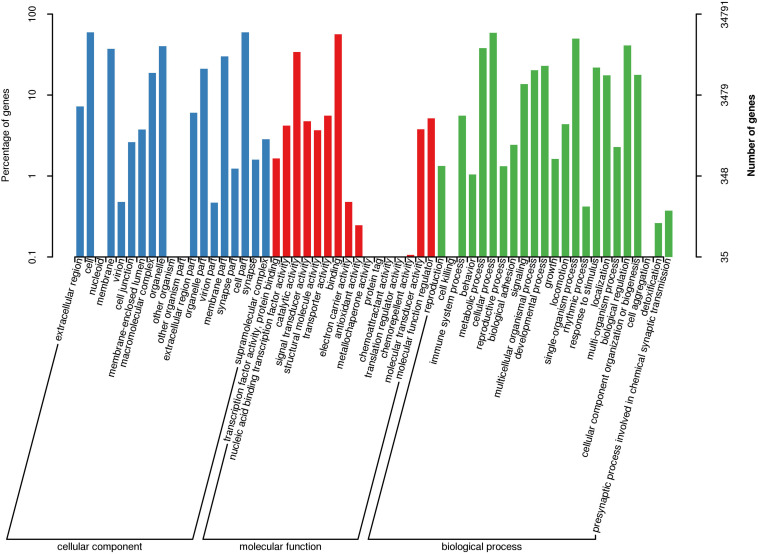
GO classification of non-redundant transcripts in bone tissues of bighead carp.

For Nr annotation, 89.69% of homologous hits were assigned to six fish species, respectively, including *Danio rerio*, *Astyanax mexicanus*, *O. mykiss*, *Esox lucius*, *Cyprinus carpio*, and *Ctenopharyngodon idella*. GO annotations generated 58 level 2 GO terms (Figure 1). Among them, the three most abundant terms under the biological process category were cellular process (20,428 transcripts), single-organism process (17,294 transcripts), and biological regulation (14,248 transcripts). Within the cellular component (CC) category, cell (20,686 transcripts), cell part (20,659 transcripts), and organelle (13,936 transcripts) were the most abundant terms. Of the 16 terms in the molecular function (MF) category, binding (19,616 transcripts), catalytic activity (11,868 transcripts), and transporter activity (1,940 transcripts) had the highest number of transcripts. Three putative growth-related GO terms, including growth (involving 565 transcripts), immune system process (involving 1,931 transcripts), and metabolic process (involving 13,193 transcripts), were successfully annotated.

A total of 69.48% annotated transcripts (30,394 of 43,747 transcripts) were annotated in the KOG database, which can be assigned to 25 categories (Supplementary Figure 1). The largest number of functional categories was signal transduction mechanisms reaching 6,286 transcripts. The following four functional categories were general function prediction only (5,947 transcripts), post-translational modification, protein turnover, chaperones (2,882 transcripts), transcription (2,325 transcripts), and function unknown (2,240 transcripts), respectively.

**FIGURE 2:**
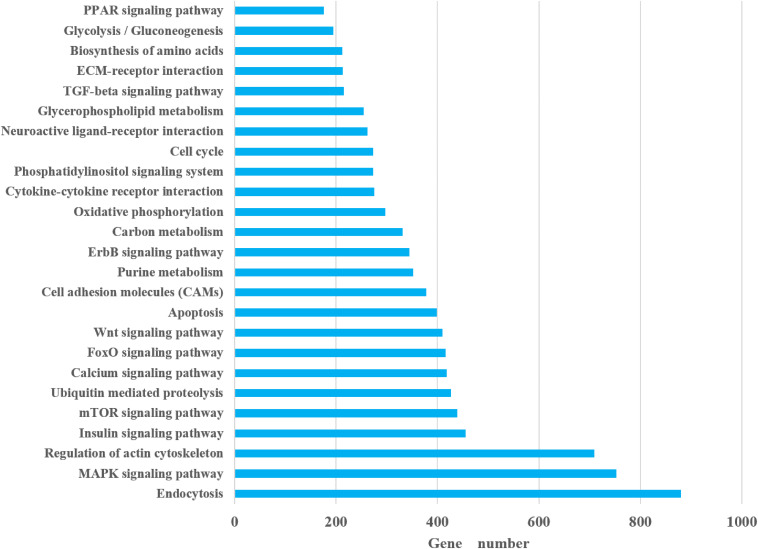
Pathways associated with growth.

In the KEGG classification, a total of 279 pathways annotated from 25,955 non-redundant transcripts were extracted from the bone transcriptome of bighead carp. The results showed that endocytosis (880 transcripts), mitogen-activated protein kinase (MAPK) signaling pathway (753 transcripts), focal adhesion (723 transcripts), regulation of actin cytoskeleton (709 transcripts), and herpes simplex infection (489 transcripts) were the top five pathways with the most abundant unigenes. Notably, we paid attention to 25 KEGG pathways (Figure 2), which may be closely associated with differential growth and involved in the physiological functions of immune, metabolism, and cytoskeleton of bighead carp, such as insulin signaling pathway, cytokine–cytokine receptor interaction, peroxisome proliferator-activated receptor (PPAR) signaling pathway, transforming growth factor β (TGF-β) signaling pathway, and regulation of actin cytoskeleton. Among these 25 KEGG pathways, endocytosis (880 unigenes), MAPK signaling pathway (753 unigenes), and regulation of actin cytoskeleton (709 unigenes) were the top three pathways with the most abundant unigenes, which provided some reference value for studying the early growth regulation and bone development in bighead carp.

### Prediction of Gene Families and Coding Sequences

We utilized the Coding Genome Reconstruction Tool (COGENT) to further partition these non-redundant transcripts into putative gene families and reconstruct each family into one or several full-length unique transcript models. Of the 63,873 non-redundant transcripts, COGENT constructed 5,474 gene families (Supplementary Table 2). A total of 44,895 ORFs (open reading frames) were identified with TransDecoder, including 34,093 complete ORFs. The length distribution of the complete coding protein sequences is shown in Supplementary Figure 2.

### *De novo* Detection of Alternative Splicing Events and Long Non-coding RNA Prediction

A total of 381 and 784 pairs of FL transcripts from F01 and F02 that might represent alternative splicing (AS) events were detected (Supplementary Table 3). Additionally, because no reference genome was available for SMRT sequencing of transcriptome in bighead carp, we could not determine the types of AS events.

**FIGURE 3:**
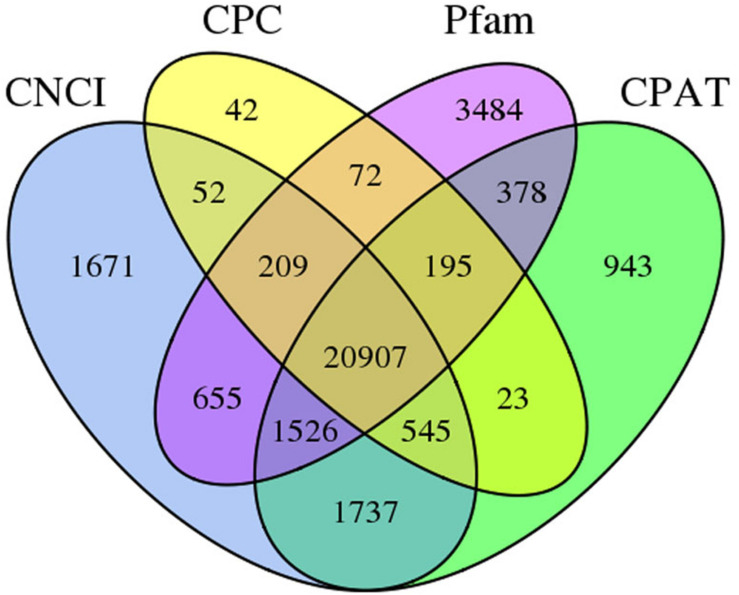
Venn graph of lncRNA transcripts from CPAT, CNCI, CPC, and Pfam analyses.

The numbers of long non-coding RNA (lncRNA) predicted from FL transcripts by Coding Potential Assessment Tool (CPAT), Coding–Non-coding Index (CNCI), Coding Potential Calculator (CPC), and Pfam were 26,254, 27,302, 22,045, and 27,426, respectively. The intersection of these four results yielded 20,907 lncRNAs (Figure 3).

### Identification of Transcription Factors and SSRs

In our Iso-Seq, a total of 1,579 non-redundant transcription factors (TF) transcripts were identified, and their detailed information is shown in Supplementary Table 4. Based on the Animal TFDB 2.0 database classification, these TFs belong to more than 56 families, and a large number of TFs were dominant in zf-C2H2, miscellaneous, and TF_bZIP (Supplementary Figure 3). It was the first time to extensively identify TFs using transcriptome dataset in bighead carp, which provided a useful foundation for TFs studies in the future.

Simple sequence repeats, also known as microsatellite DNAs, have a tandem repeat motif of 1–6 bp in length. The characters of high polymorphism (mainly due to the differences in the number of tandem motifs), stability, and reliability for SSR enable it to be an ideal molecular marker that is widely used in genetic map construction, QTL mapping, and genetic diversity assessment. We searched for SSRs in the 50,694 bighead carp transcripts longer than 500 bp, and a total of 53,508 SSRs were identified, with 13,270 transcripts containing more than one SSR. Most of the SSRs identified were mononucleotide repeats (31,758, 59.35%), followed by the dinucleotide repeats (15,540, 29.04%), trinucleotide repeats (4,989, 9.32%), tetranucleotide repeats (1,009, 1.89%), hexanucleotide repeats (199, 0.37%), and pentanucleotide repeats (13, 0.02%). Among the 53,508 SSRs, 9,085 SSRs presented in compound formation. All SSRs and their primers are listed in Supplementary Table 5.

### Identification of DEGs From Bone Tissues

To capture transcript-level expression differences related to different growth rates of bighead carp, the Illumina RNA-seq data of bone tissues, including the frontal and parietal bone and vertebra tissues, were aligned to the refined full-length transcripts of the Iso-Seq database for quantification. The DEGs of the frontal and parietal bone and vertebra tissues between the big and SGs were explored by using DEseq2 with the criteria of | log2 ratio| ≥ 1 and FDR (false discovery rate) < 0.01. Supplementary Table 6 was used as an input file to run DESeq2. In total, the numbers of DEGs from the frontal and parietal bone and vertebra tissues between BG and SG were as follows: 27 from the frontal and parietal bone tissues (15 up- and 12 down-regulated in BG) and 45 from vertebra tissues (24 up- and 21 down-regulated in BG). When we compared DEGs from bone tissues, 12 DEGs were shared in the frontal and parietal bones and vertebral bones between BG and SG, which were mainly associated with metabolic process, biological regulation, and immune system process, such as Golgi apparatus protein 1 (*glg1*), serine/threonine-protein kinase Sgk1(*sgk1*), and β2-microglobulin (*b2m*).

**FIGURE 4:**
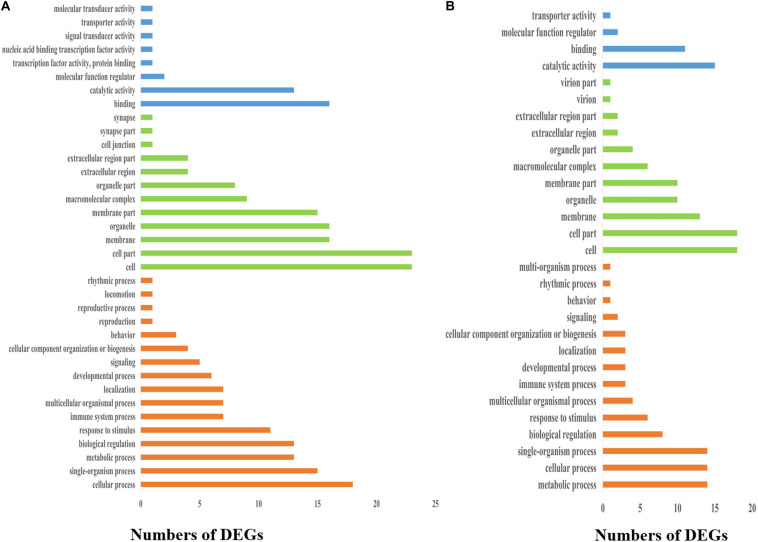
Distribution of GO classifications of DEGs in bone tissues of bighead carp. **(A)** Distribution of GO classifications of DEGs in the frontal and parietal bones. **(B)** Distribution of GO classifications of DEGs in the vertebral bones. Blue, green, and orange colors represented molecular function, cellular component, and biological process categories, respectively.

To elucidate the biological events of the DEGs from the frontal and parietal bone and vertebra tissues, which would be mainly involved in different growth rates of bighead carp at early growth stage, GO term enrichment analyses were conducted. First, GO terms were obtained on the basis of numbers of the DEGs assigned to each GO term (Figure 4). The result was as follows: Under the CC category in both the frontal and parietal bone and vertebra tissues, cell (GO: 0005623) and cell part (GO: 0044464) had the most abundant GO function items. Within the biological process (BP) category in both the frontal and parietal bone and vertebra tissues, higher percentages of genes were commonly clustered into cellular process (GO: 0009987), biological regulation (GO: 0065007), and metabolic process (GO: 0008152). In the MF category in both the frontal and parietal bone and vertebra tissues, most genes were assigned to binding (GO: 0005488), catalytic activity (GO: 0003824), and MF regulator (GO: 0003674). Second, GO terms of the DEGs were also identified according to top three *p* values of CC, BP, and MF categories, respectively. Under the CC category, the top three GO terms in both the frontal and parietal bone and vertebra tissues were extracellular exosome [GO: 0070062, *p* value = 7.4E-05 (frontal and parietal bone)/*p* value = 7.7E-05(vertebra)], endoplasmic reticulum membrane [GO: 0005789, *p* value = 0.00026 (frontal and parietal bone)/*p* value = 0.00027 (vertebra)], and mitochondrial membrane part [GO: 0044455, *p* value = 0.00044 (frontal and parietal bone)/*p* value = 0.00044 (vertebra)]. Within the BP category in both the frontal and parietal bone and vertebra tissues, translation [GO: 0006412, *p* value = 1.8E-13 [frontal and parietal bone)/*p* value = 1.9E-13(vertebra)], antigen processing and presentation [GO: 0019882, *p* value = 0.00026 (frontal and parietal bone)/*p* value = 0.00012 (vertebra)], and immune response [GO: 0006955, *p* value = 0.00478 (frontal and parietal bone)/*p* value = 0.00374 (vertebra)] were the top three GO terms. Protein serine/threonine kinase activity [GO: 0004674, *p* value = 0.00126 (frontal and parietal bone)/*p* value = 0.00128 (vertebra)], carbohydrate binding [GO: 0030246, *p* value = 0.00354 (frontal and parietal bone)/*p* value = 0.00356 (vertebra)], and monosaccharide binding [GO: 0048029, *p* value = 0.01207 (frontal and parietal bone)/*p* value = 0.01209 (vertebra)] were the top three GO terms of MF category in both the frontal and parietal bone and vertebra tissues. These common GO terms of DEGs from these two tissues might suggest the frontal and parietal bone, and vertebra tissues played roles in differential growth of bighead carp cooperatively.

**FIGURE 5:**
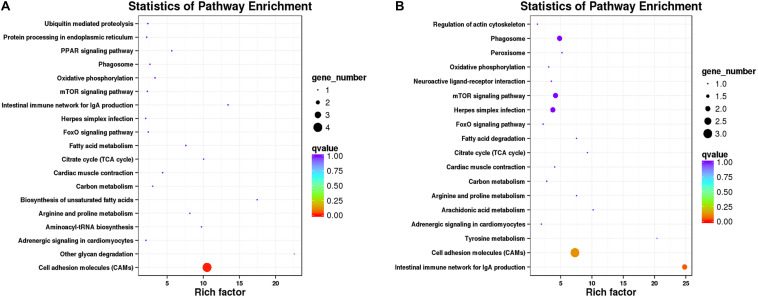
Pathway enrichment analysis of DEGs in bone tissues of bighead carp. **(A)** Enriched pathways from frontal and parietal bones between big and small groups of bighead carp. **(B)** Enriched pathways from vertebral bones between big and small groups of bighead carp.

Kyoto Encyclopedia of Genes and Genomes pathway–based analyses help to identify the biological pathways that are related to DEGs. Pathway enrichment analyses identified the enriched pathways from frontal and parietal bone and vertebra tissues, respectively (Figure 5). Among these pathways, metabolic pathways were the most frequently represented pathways in bone tissues of bighead carp with different growth rates, followed by organismal systems, environmental information processing, and genetic information processing pathways. These observations disclosed the vital implications of energy metabolism, cytoskeleton, and immune regulation in bone tissues between BG and SG of bighead carp, such as citrate cycle (TCA cycle) [ko00020, *q* = 1 (frontal and parietal bone)/*q* = 1(vertebra)], regulation of actin cytoskeleton [ko04810, *q* = 1 (vertebra)], and cell adhesion molecules (CAMs) [ko04514, *q* = 0.00761 (frontal and parietal bone)/*q* = 0.12807(vertebra)] pathways. These enriched pathways were not all significant because the number of DEGs was too few, which were considered as potential pathways associated with differential growth of bighead carp at early growth stage.

### Key DEGs Related to Differential Growth

**FIGURE 6:**
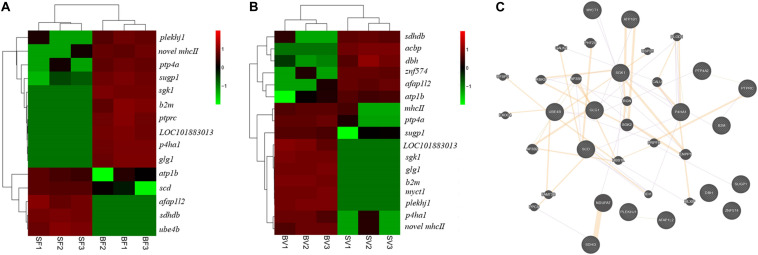
Key genes related to early growth regulation in bone tissues of bighead carp. **(A)** Key genes related to early growth regulation in the frontal and parietal bones. **(B)** Key genes related to early growth regulation in the vertebral bones. The FPKM data of genes was used for heatmap construction. **(C)** The correlation network of key genes. The round nodes filled with diagonal lines indicate key genes related to early growth regulation in bone tissues. The round nodes filled with pure color indicate genes that interact with key genes. The interactions among different genes were represented by different colorful lines. Orange lines indicate predicted interactions. Purple lines indicate co-expression interactions. Gene abbreviations: pleckstrin homology domain-containing family J member 1 (*plekhj1*), SURP and G-patch domain-containing protein 1 (*sugp1*), prolyl 4-hydroxylase subunit alpha-1 (*p4ha1*), novel MHCII β chain protein (*novel mhc II*), protein tyrosine phosphatase type IVA (*ptp4a*), β2-microglobulin (*b2m*), serine/threonine-protein kinase Sgk1 (*sgk1*), receptor-type tyrosine-protein phosphatase C (*ptprc*), uncharacterized protein LOC101883013 (*LOC101883013*), myc target protein 1(*myct1*), golgi apparatus protein 1 (*glg1*), sodium/potassium-transporting ATPase subunit β (*atp1b*), stearoyl-CoA desaturase (*scd*), dopamine β-hydroxylase (*dbh*), succinate dehydrogenase [ubiquinone] cytochrome b small subunit B (*sdhdb*), MHC class II β precursor (*mhcII*), actin filament–associated protein 1-like 2 (*afap1l2*), acyl-CoA–binding protein (*acbp*), ubiquitin conjugation factor E4 B (*ube4b*), zinc finger protein 574 (*znf574*).

In total, 15 key pathways associated with the early growth regulation of bighead carp were identified from frontal and parietal bone and vertebra tissues (Table 3), which played important roles in the physiological functions of energy metabolism, cytoskeleton, and immune regulation. Twenty key DEGs (Figure 6) were selected by three strategies simultaneously, which were considered to be related to the differential growth of bighead carp: (i) 10 important DEGs that showed high participation frequencies in the 15 key pathways; (ii) another seven important DEGs that appeared more than two times in biological regulation, immune system process, and metabolic process of GO term enrichment analyses; (iii) three important DEGs that were commonly identified in frontal and parietal bone and vertebra tissues. Then, these 20 key DEGs were used to construct the correlation network by GeneMANIA (Figure 6).

**TABLE 3:** Key pathways related to early growth regulation in bone tissues of bighead carp.

**Pathway**	**Ko Id**	**DEGs**	**Tissue**	**Expression tendency**	**FDR**
Adrenergic signaling in cardiomyocytes	ko04261	*atp1b1*	F	Down	0.00408
			V	Down	0.00228
Arginine and proline metabolism	ko00330	*p4ha1*	F	Up	0.00018
			V	Up	0.00018
Biosynthesis of unsaturated fatty acids	ko01040	*scd*	F	Down	2.23E-06
Carbon metabolism	ko01200	*sdhdb*	F	Down	2.77E-09
			V	Down	5.97E-07
Cardiac muscle contraction	ko04260	*atp1b1*	F	Down	0.00408
			V	Down	0.00228
Cell adhesion molecules (CAMs)	ko04514	*b2m*	F	Up	0.00075
			V	Up	1.41E-19
		*glg1*	F	Up	0.00049
			V	Up	3.77E-07
		*mhc II*	F	Up	0.00165
			V	Up	0.00172
		*ptprc*	F	Up	0.00083
Citrate cycle (TCA cycle)	ko00020	*sdhdb*	F	Down	2.77E-09
			V	Down	5.97E-07
Fatty acid metabolism	ko01212	*scd*	F	Down	2.23E-06
FoxO signaling pathway	ko04068	*sgk1*	F	Up	0.00534
			V	Up	7.32E-11
Intestinal immune network for IgA production	ko04672	*b2m*	F	Up	0.00075
			V	Up	1.41E-19
		*mhcII*	F	Up	0.00165
			V	Up	0.00172
mTOR signaling pathway	ko04150	*lamtor1*	V	Down	0.00066
		*sgk1*	F	Up	0.00534
			V	Up	7.32E-11
Oxidative phosphorylation	ko00190	*sdhdb*	F	Down	2.77E-09
			V	Down	5.97E-07
PPAR signaling pathway	ko03320	*scd*	F	Down	2.23E-06
Regulation of actin cytoskeleton	ko04810	*arhgef6*	V	Up	0.00538
Phagosome	ko04145	*b2m*	F	Up	0.00075
			V	Up	1.41E-19
		*mhcII*	F	Up	0.00165
			V	Up	0.00172
Pathway	Ko Id	DEGs	Tissue	Expression Tendency	FDR
Adrenergic signaling in cardiomyocytes	ko04261	*atp1b1*	F	Down	0.00408
			V	Down	0.00228
Arginine and proline metabolism	ko00330	*p4ha1*	F	Up	0.00018
			V	Up	0.00018
Biosynthesis of unsaturated fatty acids	ko01040	*scd*	F	Down	2.23E-06
Carbon metabolism	ko01200	*sdhdb*	F	Down	2.77E-09
			V	Down	5.97E-07
Cardiac muscle contraction	ko04260	*atp1b1*	F	Down	0.00408
			V	Down	0.00228
Cell adhesion molecules (CAMs)	ko04514	*b2m*	F	Up	0.00075
			V	Up	1.41E-19
		*glg1*	F	Up	0.00049
			V	Up	3.77E-07
		*mhc II*	F	Up	0.00165
			V	Up	0.00172
		*ptprc*	F	Up	0.00083
Citrate cycle (TCA cycle)	ko00020	*sdhdb*	F	Down	2.77E-09
			V	Down	5.97E-07
Fatty acid metabolism	ko01212	*scd*	F	Down	2.23E-06
FoxO signaling pathway	ko04068	*sgk1*	F	Up	0.00534
			V	Up	7.32E-11
Intestinal immune network for IgA production	ko04672	*b2m*	F	Up	0.00075
			V	Up	1.41E-19
		*mhcII*	F	Up	0.00165
			V	Up	0.00172
mTOR signaling pathway	ko04150	*lamtor1*	V	Down	0.00066
		*sgk1*	F	Up	0.00534
			V	Up	7.32E-11
Oxidative phosphorylation	ko00190	*sdhdb*	F	Down	2.77E-09
			V	Down	5.97E-07
PPAR signaling pathway	ko03320	*scd*	F	Down	2.23E-06
Regulation of actin cytoskeleton	ko04810	*arhgef6*	V	Up	0.00538
Phagosome	ko04145	*b2m*	F	Up	0.00075
			V	Up	1.41E-19
		*mhcII*	F	Up	0.00165
			V	Up	0.00172

*In the “Tissue” column, F is the abbreviation of the frontal and parietal bone, and V means the abbreviation of vertebra. Gene abbreviations: sodium/potassium-transporting ATPase subunit β (*atp1b*), prolyl 4-hydroxylase subunit alpha-1 (*p4ha1*), stearoyl-CoA desaturase (*scd*), succinate dehydrogenase [ubiquinone] cytochrome b small subunit B (*sdhdb*), β2-microglobulin (*b2m*), golgi apparatus protein 1 (*glg1*), MHC class II β precursor (*mhcII*), receptor-type tyrosine-protein phosphatase C (*ptprc*), serine/threonine-protein kinase Sgk1 (*sgk1*), regulator complex protein LAMTOR1 (*lamtor1*), Rho guanine nucleotide exchange factor 6 (*arhgef6*).*

### Validation of DEGs by Quantitative Real-Time Polymerase Chain Reaction

**FIGURE 7:**
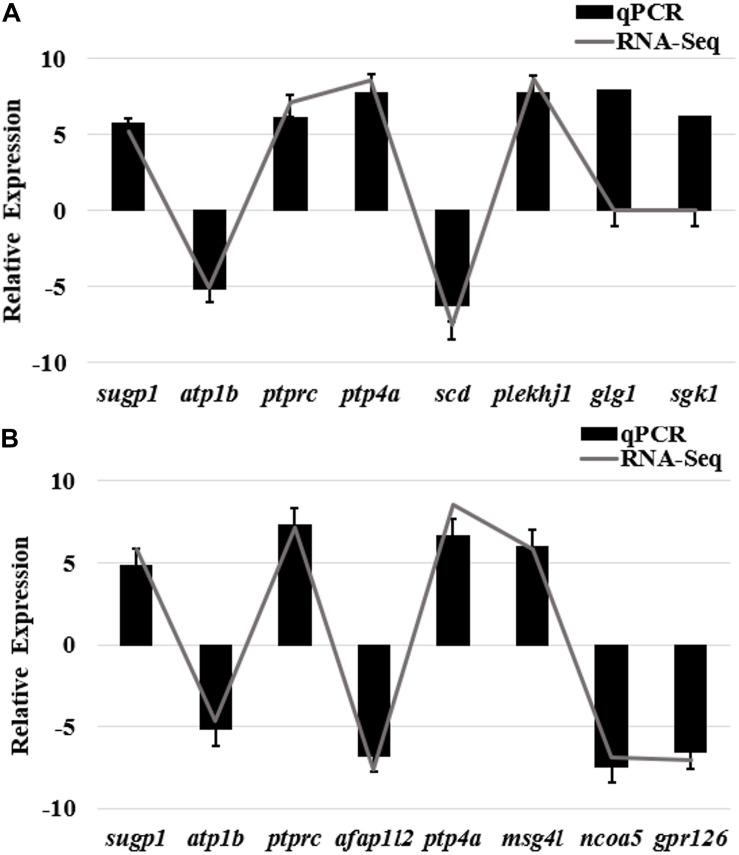
qRT-PCR validation of differently expressed genes in bone tissues of bighead carp from differential growth groups. **(A)** qRT-PCR validation of differently expressed genes of frontal and parietal bones between big and small groups of bighead carp. **(B)** qRT-PCR validation of differently expressed genes of vertebral bones between big and small groups of bighead carp. Gene expressions were expressed as mean normalized ratios (*n* = 3, ± SE). β*-Actin* was used as reference gene. Positive values denote up-regulation in big group compared to small group of bighead carp. Gene abbreviations: SURP and G-patch domain-containing protein 1 (*sugp1*), sodium/potassium-transporting ATPase subunit β (*atp1b*), receptor-type tyrosine-protein phosphatase C (*ptprc*), protein tyrosine phosphatase type IVA (*ptp4a*), stearoyl-CoA desaturase (*scd*), pleckstrin homology domain-containing family J member 1 (*plekhj1*), actin filament–associated protein 1-like 2 (*afap1l2*), microfibril-associated glycoprotein 4-like (*msg4l*), nuclear receptor co-activator 5 (*ncoa5*), golgi apparatus protein 1 (*glg1*), G-protein coupled receptor 126(*gpr126*), serine/threonine-protein kinase Sgk1(*sgk1*).

Quantitative real-time polymerase chain reaction (qRT-PCR) was performed on 12 selected genes and the internal control gene β*-actin* (Supplementary Table 7), to validate the DEGs identified in bone tissues of bighead carp by RNA-seq. Fold changes from qRT-PCR were compared with the RNA-seq expression profiles (Figure 7). The expression patterns revealed by qRT-PCR analysis were similar to those revealed by RNA-seq for the same gene. Thus, RNA-seq could provide reliable data for mRNA differential expression analyses.

## Discussion

Transcriptome reconstruction and annotation have significantly improved with the advent of new sequencing technologies. These techniques play an important role in gene discovery, genome annotation, and detection of genomic signatures, particularly in species without a reference genome (Li et al., 2017). In recent years, traditional short-read RNA-seq has been commonly used to investigate RNA expression patterns in several tissues such as that of the brain, liver, and muscle (Zhou et al., 2019; Cleveland et al., 2020). However, short-read RNA-seq has certain limitations in regard to precise reconstruction and reliable sequencing of isoforms due to the complexity of AS mechanisms in eukaryotes (Tilgner et al., 2013; Yi et al., 2018). In contrast, SMRT sequencing is a superior strategy, directly generating a comprehensive transcriptome with accurate sequencing of AS isoforms and novel genes, and its advantages have been extensively documented in previous studies. In this study, the full-length transcriptome of bighead carp was performed using the Iso-Seq technique.

Previously reported transcriptomic studies of the bighead carp were performed using Illumina sequencing platforms (Fu B. et al., 2019; Li et al., 2019). In comparison, our study produced a comprehensive transcriptome with several features. First, accurate full-length transcripts (63,872 transcripts) were generated, serving as a valuable resource for various gene structures and sequences, which can be directly used in gene-function studies without using additional gene clones. Second, a total of 20,907 lncRNAs were identified, which could be useful for investigations of potential lncRNA functions in the bighead carp. Third, the full-length transcripts could serve as a reference for genome assembly and gene annotation of bighead carp. Finally, reliable ORFs identified in this study are essential for the identification of orthologous genes, aiding in the better understanding of culture and breeding techniques used for bighead carp.

A total of 279 pathways were annotated from 25,955 non-redundant transcripts extracted from the bone transcriptome of the bighead carp, using the KEGG classification system. Notably, we focused on 25 KEGG pathways (Figure 2), which are potentially associated with regulation of early growth and are involved in physiological functions of the immune system, metabolism, and cytoskeleton of bighead carp. These KEGG pathways included the insulin signaling pathway, cytokine–cytokine receptor interaction, PPAR signaling pathway, TGF-β signaling pathway, Wnt signaling pathway, and regulation of actin cytoskeleton and were also the focus of previous reports on growth and bone development (Nie et al., 2019; Cleveland et al., 2020), suggesting that they play important roles in growth modulation and bone development of bighead carp at early growth stage.

To identify growth-related genes in the bighead carp, the DEGs in the big and the SGs were identified and functionally analyzed. In total, 15 key enriched pathways (Table 3) and 20 key DEGs (Figure 6), of the frontal and parietal bones and the vertebra, were screened and found to be mainly involved in the physiological functions of metabolism, cytoskeleton, and immunization. Arginine and proline metabolism, biosynthesis of unsaturated fatty acids, PPAR signaling pathway, citrate cycle, oxidative phosphorylation pathway, and carbon and fatty acid–metabolizing pathways are responsible for metabolizing amino acids, lipids, and carbohydrates, producing energy for vital functions of an organism (Jiao et al., 2020). Most of these metabolic pathways are also found in reports of growth-related transcriptomes in fish, such as the PPAR signaling pathway (Lu et al., 2019), carbon-metabolizing pathways (Lu et al., 2020), and oxidative phosphorylation pathway (Fu J. et al., 2019). The eight other aforementioned pathways also play an important role in growth, by regulating the actin cytoskeleton, cell adhesion molecules, and FoxO signaling pathway (Pang et al., 2018; Lu et al., 2019).

Previous studies have shown that energy metabolism can influence the growth of an organism (Lu et al., 2019). In this study, DEGs, potentially influencing amino acid, lipid, and carbohydrate metabolism of bighead carp, were identified using transcriptome analysis. For instance, acyl-CoA–binding protein (*acbp*), *SURP*, G-patch domain-containing protein 1 (*sugp1*), and stearoyl-CoA desaturase (*scd*) are important lipogenic enzymes, and a change in their protein activities can alter the rates of biosynthesis of fatty acids (Igal, 2016; Kim et al., 2016; Zhou et al., 2020). Succinate dehydrogenase [ubiquinone] cytochrome b small subunit B (*sdhdb*) plays an important role in lipid transport and metabolism, as well as in carbon metabolism (Oyedotun and Lemire, 1999). Prolyl 4-hydroxylase subunit alpha-1 (*p4ha1*) and ubiquitination factor E4B (*ube4b*) are involved in protein synthesis and degradation (Chakravarthi et al., 2014; Lu et al., 2019). Moreover, several immune-related DEGs were identified in the bone tissue transcriptome of bighead carp, such as β2-microglobulin (*b2m*), MHC class II β precursor, receptor-type tyrosine-protein phosphatase C (*ptprc*), and dopamine β-hydroxylase (*dbh*) (Kountikov et al., 2004; Das et al., 2011; Cheng et al., 2017). It has been previously shown that these immune system-related genes are essential for maintaining normal growth and physiological functions (Cleveland et al., 2020). In addition to the genes of energy metabolism and immune function, cytoskeletal genes have been identified as candidate growth-related genes in fish (Salem et al., 2012). In this study, we found a differential expression of cytoskeletal genes such as myc target protein 1 homolog (*myct1*), Golgi apparatus protein 1 (*glg1*), zinc finger protein 574 (*znf574*), and actin filament–associated protein 1-like 2 (*afap1l2*) in the two groups with different growth rates (Mourelatos et al., 1995; Berg et al., 2010; Tie et al., 2016; Wu et al., 2016), suggesting that they play important roles in cell growth, proliferation, apoptosis, and transformation. The protein tyrosine phosphatase 4A (PTP4A) family, consisting of PTP4A1/PRL1, PTP4A2/PRL2, and PTP4A3/PRL3, has been implicated in cell proliferation and tumorigenesis (Kobayashi et al., 2014). *Ptp4a* is a critical promoter of TGF-β signaling pathway in primary dermal fibroblasts (Sacchetti et al., 2017) and might also regulate the growth of the bighead carp. Serine/threonine-protein kinase (*Sgk1*) is a serum glucocorticoid kinase that is involved in the regulation of fat storage, body size, and development in *Caenorhabditis elegans* (Jones et al., 2009). *Sgk1* has also been identified as a growth-related gene in the Arctic charr (*Salvelinus alpinus*), at a specific developmental stage, and is correlated to the size of the organism (Beck et al., 2019). Although reports regarding DEGs pleckstrin homology domain-containing family J member 1 (*plekhj1*) and uncharacterized protein (*LOC101883013*) are rare, we consider them as novel candidate growth-related genes that need further functional investigation and verification.

Among the aforementioned DEGs (Figure 6), several DEGs have been identified as candidate genes regulating growth in previous transcriptomic and genomic studies, including *scd*, *ube4b*, *atp1b*, *myct1*, *b2m*, and *mhcII* (Xu et al., 2013; Wu et al., 2016; Lu et al., 2019; Zhou et al., 2019). These reports provide additional support to our findings of these genes being significantly involved in the modulation of differential growth in different domestic animals. However, compared with other transcriptomes of bone tissues in fish, certain well-known pathways and genes involved in bone formation and differentiation have not been identified in DEG analyses of frontal and parietal bones and vertebral bones in bighead carp, such as calcium, MAPK, TGF-β, and osteoclast pathways (Lu et al., 2019; Nie et al., 2019). For our study, this may be because the bighead carp used were 6 months old, and bone formation and differentiation might have already been completed. Bone formation and differentiation in fish usually occur during early growth and development, approximately 30 days post-fertilization or even earlier, such as in *Epinephelus lanceolatus* (Lv et al., 2019), *M. amblycephala* (Nie et al., 2019), and *Cynoglossus semilaevis* (Ma et al., 2019). Although well-known pathways and genes involved in bone formation and differentiation were not identified in this study, we found several growth-related DEGs that have been previously reported to regulate bone development, such as *p4ha1* (Zou et al., 2017) and *sgk1* (Beck et al., 2019), suggesting that differential growth of bighead carp is associated with bone development, to an extent.

## Materials and Methods

### Ethics Statement

All experimental protocols involved in fish in this study were conducted in strict accordance with the recommendations in the Guide for the Care and Use of Laboratory Animals of the Institute of Hydrobiology, the Chinese Academy of Sciences, China. All efforts were made to minimize suffering of the fish.

### Fish Sample Collection

**FIGURE 8:**
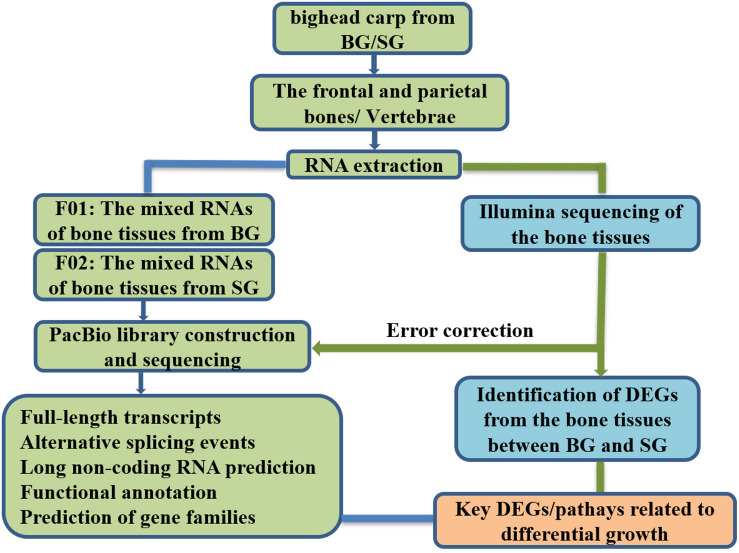
The layout plan of the study design. BG is the abbreviation of big bighead carp, and SG means small bighead carp.

Fish samples of bighead carp at early growth stage used in this study originated from one full-sib family, which were cultured at one pond of the Zhangdu Lake Fish Farm (Wuhan, China). In November 2018, BL, HL, HH, HW, and BW of 6-month samples were measured after being anesthetized with MS-222 (tricaine methanesulfonate; Sigma). The ratios of HL and BL (HL/BL) were calculated. Three samples with larger BW were clustered in fast-growing group (BG). Another three samples with smaller BW were clustered in slow-growing group (SG). The frontal and parietal bones were separated from the skull, removed skins, washed with pure water, cut into pieces, mixed together, and then immediately placed in liquid nitrogen and stored at −80°C refrigerator before total RNA extraction. The vertebrae were separated from the body, removed muscles, also washed with pure water, cut into pieces, mixed, and then immediately placed in liquid nitrogen and stored at −80°C refrigerator before total RNA extraction. The layout plan of the study design is shown in Figure 8.

### RNA Extraction and Quality Evaluation

Total RNA was extracted from each bone tissue (the frontal and parietal bones or vertebral bones) using RNAiso reagent (Takara, Tokyo, Japan). To prevent genomic DNA contamination, RNA samples were treated to digest DNA using RNase-free DNase I during extraction of total RNA. RNA degradation and contamination were verified by ethidium bromide staining of 28s and 18s ribosomal RNA on a 1% agarose gel. RNA purity and concentration were checked using a Nanodrop 2000 spectrophotometer (Thermo Scientific). RNA integrity was assessed using an Agilent RNA 6000 Nano reagents part I Kit in an Agilent 2100 Bioanalyzer System (Agilent Technologies, Santa Clara, CA, United States). The RNA quality criteria for the RNA samples were RIN ≥ 8.0 (RNA Integrity Number), and 1.8 < OD260/280 < 2.2. The qualified RNAs were used for further PacBio and Illumina library construction, respectively.

### PacBio Library Construction and Sequencing

To construct the library for PacBio sequencing, the qualified RNAs of bone tissues from BG and SG, including the frontal and parietal bone and vertebra tissues, were mixed in equal amounts, respectively. The mixed RNA sample from BG or SG was reverse-transcribed for mRNA using the SMARTer^TM^ PCR cDNA Synthesis Kit. PCR amplification was performed using the KAPA HiFi HotStart PCR Kit. Then, the PCR product for the SMRTbell library was constructed using the SMRTbell template pre kit. The concentration of the SMRTbell library was measured using a Qubit 3.0 fluorometer with a Qubit^TM^ 1× dsDNA HS Assay kit (Invitrogen, Carlsbad, CA, United States). The quantified criteria of library quality were concentration >10 ng/μL with dispersive but continuous distribution in the range of 1–6 k bp. A total of 2.5 ng of the library was sequenced for each SMRT cell using the binding kit 2.1 from the PacBio Sequel platform, producing 20 h of movies. In the sequencing process, two SMRT cells were prepared on the PacBio RSII platform, including one SMRT cell from mixed bone tissues in BG and the other SMRT cell from bone tissues in SG.

### Illumina RNA-Seq and *de novo* Assembly

The Illumina library for each tissue sample was constructed using the TruSeq RNA Sample Prep Kit (Illumina) following the manufacturer’s instructions. Briefly, the polyA mRNA was fragmented using divalent cations at elevated temperature. The RNA fragments were reverse transcribed into first-strand cDNA using reverse transcriptase and random primers, followed by second-strand cDNA synthesis, end repair, and ligation of the adapters. The ligated fragments were purified and enriched through PCR to generate the final cDNA library. Finally, 12 transcriptomic libraries (six libraries from each group) were sequenced on Illumina HiSeq × Ten platform to obtain 150 bp pair-end reads. Raw RNA-seq reads in fastq format were first filtered through in-house perl scripts to filter out the low-quality reads. Reads with a Q30 percentage greater than 85% were retained as high-quality reads; the rest of the reads as low-quality reads were filtered out. Then clean reads were obtained by removing reads containing sequencing adapters, ploy-N, and low quality. The clean paired-end reads from each library were merged together and then *de novo* assembled by using Trinity 2.8.4 software with the default parameters. The clean short reads were then mapped to the PacBio reference sequence using Tophat2 tools.

### PacBio Iso-Seq Data Processing and Error Correction

According to PacBio’s protocol, the raw polymerase reads were first processed using SMRTlink 5.0 software. Briefly, after removing the SMRTbell^TM^ adapter and the low-quality data, postfilter polymerase reads were obtained. The CCS was generated from the subreads BAM files, also known as the reads of insert (ROIs). All the ROIs whose the number of full passes was >1 were further classified into full-length (FL) and non–full-length (nFL) transcript sequences based on whether the 5′ primer, 3′ primer, and poly A tail could be simultaneously observed. We employed a three-step strategy for error correction to improve the accuracy of the full-length transcripts produced by the PacBio Iso-Seq platform. First, the circle sequencing with >1 pass provided an opportunity for ROI self-correction. Second, full-length, non-chimeric (FLNC) reads were subjected to non-redundant and clustering treatments by the ICE Quiver algorithm and to arrow polishing with the nFL sequence, producing high-quality and polished full-length consensus sequences. Finally, these polished consensus sequences were further subjected to correction and redundancy removal with Illumina short reads using the Proovread tool and the CD-HIT program with a–c 0.99 parameter cutoff, respectively. The above three corrections resulted in non-redundant, non-chimeric, full-length transcripts (isoform level) with high accuracy for subsequent analyses.

### Functional Annotation

For comprehensive functional annotation, the transcripts were searched against seven databases using BLAST software (version 2.2.26): NCBI NR, COG, Pfam, KOG, Swiss-Prot (A manually annotated and reviewed protein sequence database), GO, and eggNOG using an *e* value of 1*e*^–5^. The KEGG orthology results were obtained by comparing with KEGG database using KOBAS2.0 (Xie et al., 2011). After predicting the amino acid sequence of transcripts, the software HMMER (Eddy, 1998) was used to compare them with Pfam database to get the annotation information of transcripts.

### Gene Family and Coding Sequence Prediction

Coding Genome Reconstruction Tool v1.3^1^ used K-mer similarity profiles to partition full-length coding sequences into gene families, after which it reconstructed subreads containing the full coding region. To predict the ORFs in transcripts, we used the TransDecoder v2.0.1 program^2^ to define putative coding sequences (CDSs). The predicted CDSs were searched and confirmed by BLASTX (*E* value < 1 × 10^–5^) against the protein databases of NR, Swiss-Prot, and KEGG. Those transcripts containing complete ORFs were designated as full-length transcripts.

### Prediction of LncRNA and AS

Transcripts with a length of more than 200 bp and having more than two exons were selected as lncRNA candidates. The lncRNAs were predicted by four computational approaches, including CNCI (v2), CPC (v1), CPAT (v1.2), and Pfam (v1.5) with default parameters. These approaches can effectively distinguish protein-coding and non-coding transcripts. Transcripts were removed that did not pass any of these analyses; the intersection of the four results was then selected as lncRNAs.

Based on the BLAST method (Altschul et al., 1997), all the transcripts were used for pairwise alignment. BLAST alignments were considered products of candidate AS events (Liu et al., 2017), which met three criteria simultaneously: (i) the length of two transcripts was both greater than 1,000 bp, and there were two high-scoring segment pairs in the alignment; (ii) the AS gap between two aligned transcripts was greater than 100 bp and at least 100 bp from the 3′ end and 5′ end; and (iii) a 5-bp overlap could be allowed.

### Detection of TF and Microsatellite Markers

Transcription factors-related transcript sequences were predicted using the BLAST method with the AnimalTFDB database (Zhang et al., 2015).

Microsatellite markers (also known as SSRs) were identified using MISA^3^ with parameters as default. SSR detection was only conducted on non-redundant transcripts that were larger than 500 bp in size. The minimum repeat time for core repeat motifs was set as follows: 10 for mononucleotide, six for dinucleotides, and five for trinucleotides, tetranucleotides, pentanucleotides, and hexanucleotides. Based on the structural organization of the repeat motifs, SSRs were classified into perfect and complicated (compound or interrupted) SSRs.

### Screening Differentially Expressed Unigenes and GO and KEGG Enrichment Analyses

The expression levels of all the unigenes in 12 samples were assayed based on the Illumina short reads dataset, and reference sequences were the full-length transcripts yielded from PacBio Iso-Seq. The transcripts were quantified using RSEM software. Relative gene expression levels of each unigene were determined by FPKM (fragments per kilobase of transcript per million mapped reads). DEGs of bone tissues (the frontal and parietal bones and vertebral bones) in BG and SG were screened using DESeq2 package. Supplementary Table 6 was used as an input file to run DESeq2. The FDR (<0.01) adjusted by Benjamini–Hochberg method was adopted for screening DEGs. DEGs were defined as by parameters of FDR < 0.01 and the absolute value of the log2 ratio ≥ 1. DEGs were also employed for the enrichment analyses of GO and KEGG pathways in order to determine the potential functions and metabolic pathways.

### Validation of Differentially Expressed Unigenes by qRT-PCR

In order to examine the reliability of the RNA-seq results, 12 DEGs randomly selected and the internal control gene β*-actin* were used for validation by qRT-PCR. Total RNA from 12 samples (each of the frontal and parietal bones and vertebrae from BG and SG) was extracted individually using TRIzol Reagent (Invitrogen) according to the manufacturer’s instruction. The cDNA was synthesized from 1 μ g of total RNA for each sample using PrimeScript^TM^ RT reagent Kit (TaKaRa, Dalian, China). The selected DEGs and specific primer sequences used for qRT-PCR are listed in Supplementary Table 7, qRT-PCR was performed on a StepOne^TM^ Real-Time PCR System (Applied Biosystems, Foster City, CA, United States). The qRT-PCR reaction solution consisted of 6.5 μL Power SYBR Green PCR Master Mix (Applied Biosystems), 0.2 μM of each forward and reverse primer, 1.2 μL diluted cDNA, and 4.5 μL sterile distilled water. The PCR reaction condition was performed at 95°C for 10 min, followed by 40 cycles of 95°C for 15 s, 58°C for 30 s, and 72°C for 45 s. RNA samples in bone tissues of bighead carp from big and SGs were run in three times’ biological replicates and three technical replicates for qRT-PCR. The expression level of each DEG was normalized to that of the reference gene β*-actin* by using the 2^–ΔΔCT^ value method (Livak and Schmittgen, 2001) to validate the results of RNA-seq.

## Conclusion

In summary, we obtained full-length transcriptome sequences of the frontal and parietal bones and vertebral bones by PacBio Iso-Seq in bighead carp at early growth stage known to exhibit different growth rates. Coupling the RNA-seq data with the Iso-Seq results of the big and the SGs, 27 and 45 DEGs were identified from skull bones and vertebral bones, respectively. A total of 15 pathways and 20 DEGs potentially regulate differential growth in bighead carp and were found to be mainly involved in physiological functions of energy metabolism, immune function, and cytoskeleton function, such as arginine and proline metabolism (*p4ha1*), fatty acid metabolism (*scd*), oxidative phosphorylation (*sdhdb*), FoxO signaling (*sgk1*), and cell adhesion molecules (*b2m*, *mhcII*, *ptprc*, and *glg1*). Our study represents the first step in establishing a full-length transcriptome resource of the head and vertebral bones in bighead carp at early growth stage and sheds light on the genetic association between growth and bone development.

## Data Availability Statement

The datasets presented in this study can be found in online repositories. The names of the repository/repositories and accession number(s) can be found below: https://www.ncbi.nlm.nih.gov/, BioProject ID PRJNA661719.

## Ethics Statement

The animal study was reviewed and approved by the Guide for the Care and Use of Laboratory Animals of the Institute of Hydrobiology, the Chinese Academy of Sciences, China.

## Author Contributions

JT conceived and designed the experiments and modified the manuscript. WL, YZ, JW, and XY performed the experiments. WL analyzed the data and wrote the manuscript. All authors read and approved the final manuscript.

## Conflict of Interest

The authors declare that the research was conducted in the absence of any commercial or financial relationships that could be construed as a potential conflict of interest.

## Footnotes

1 https://github.com/Magdoll/Cogent

2 https://transdecoder.github.io/

3 http://pgrc.ipk-gatersleben.de/misa/misa.html

## Supplementary Material

Supplementary Figure 1 KOG classification of non-redundant transcripts in bone tissues of bighead carp.

Supplementary Figure 2 The length distribution of the complete coding protein sequences in bone tissues of bighead carp.

Supplementary Figure 3 Type distribution of transcription factors in bone tissues of bighead carp.

Supplementary Table 1 Summary statistics for RNA-seq information of the bighead carp frontal and parietal bone and vertebra transcriptomes.

Supplementary Table 2 Putative gene families of non-redundant transcripts in bone tissues of bighead carp.

Supplementary Table 3 Alternative splicing of non-redundant transcripts in bone tissues of bighead carp.

Supplementary Table 4 Detailed information of transcription factors in bone tissues of bighead carp.

Supplementary Table 5 Information of SSRs and their primers in bone tissues of bighead carp.

Supplementary Table 6 An input file to run DESeq2 for screening DEGs of bighead carp.

Supplementary Table 7 Primer sequences of the DEGs for qRT-PCR in bone tissues of bighead carp.
