# Supplementary figures and images for: Identifying Candidate Genes Involved in the Regulation of Early Growth Using Full-Length Transcriptome and RNA-Seq Analyses of Frontal and Parietal Bones and Vertebral Bones in Bighead Carp (Hypophthalmichthys nobilis)
Source: Front Genet. 2021 Jan 15;11:603454. doi: 10.3389/fgene.2020.603454 (PMC7844397; doi:10.3389/fgene.2020.603454)

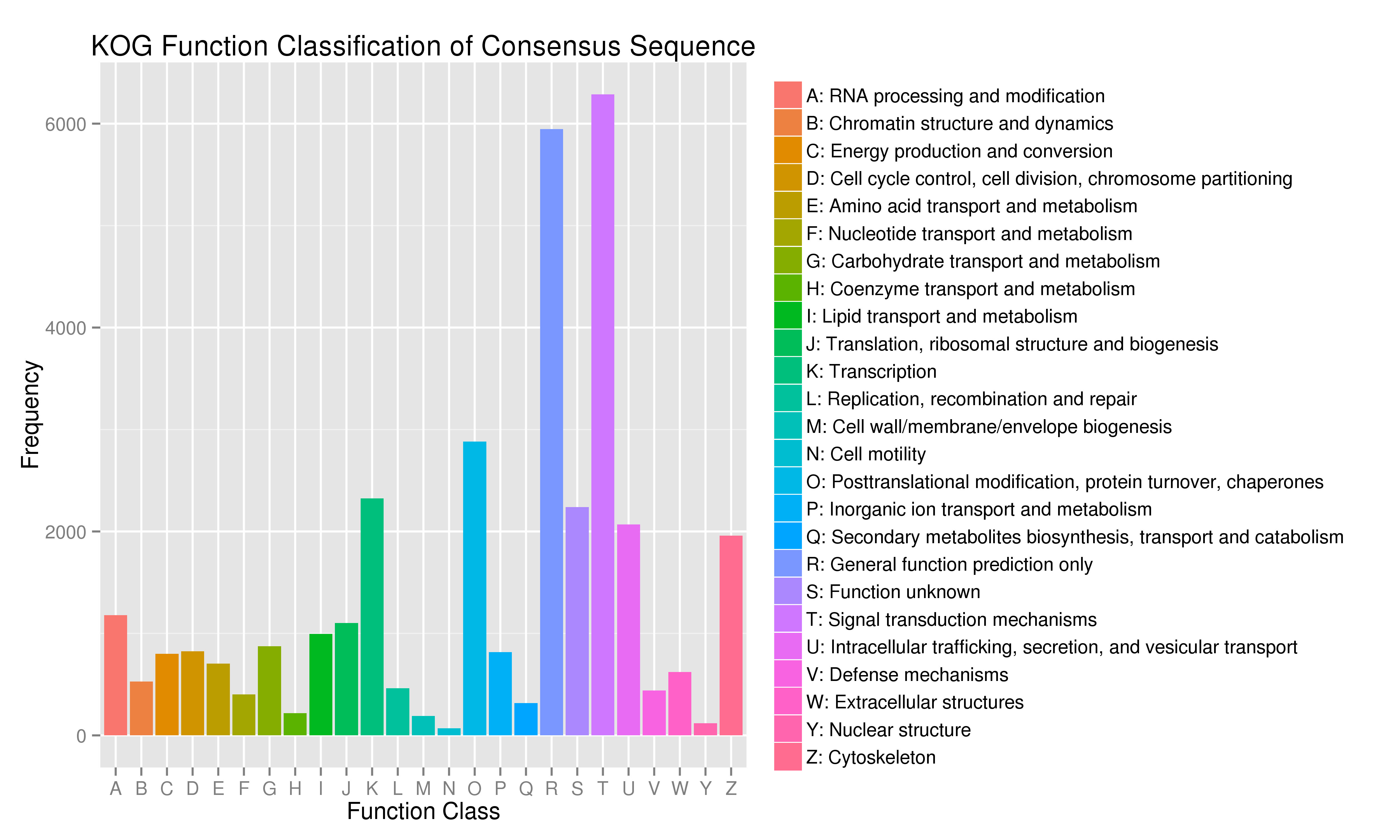

Supplement: Supplementary Figure 1 — KOG classification of non-redundant transcripts in bone tissues of bighead carp. [file Image_1.JPEG]

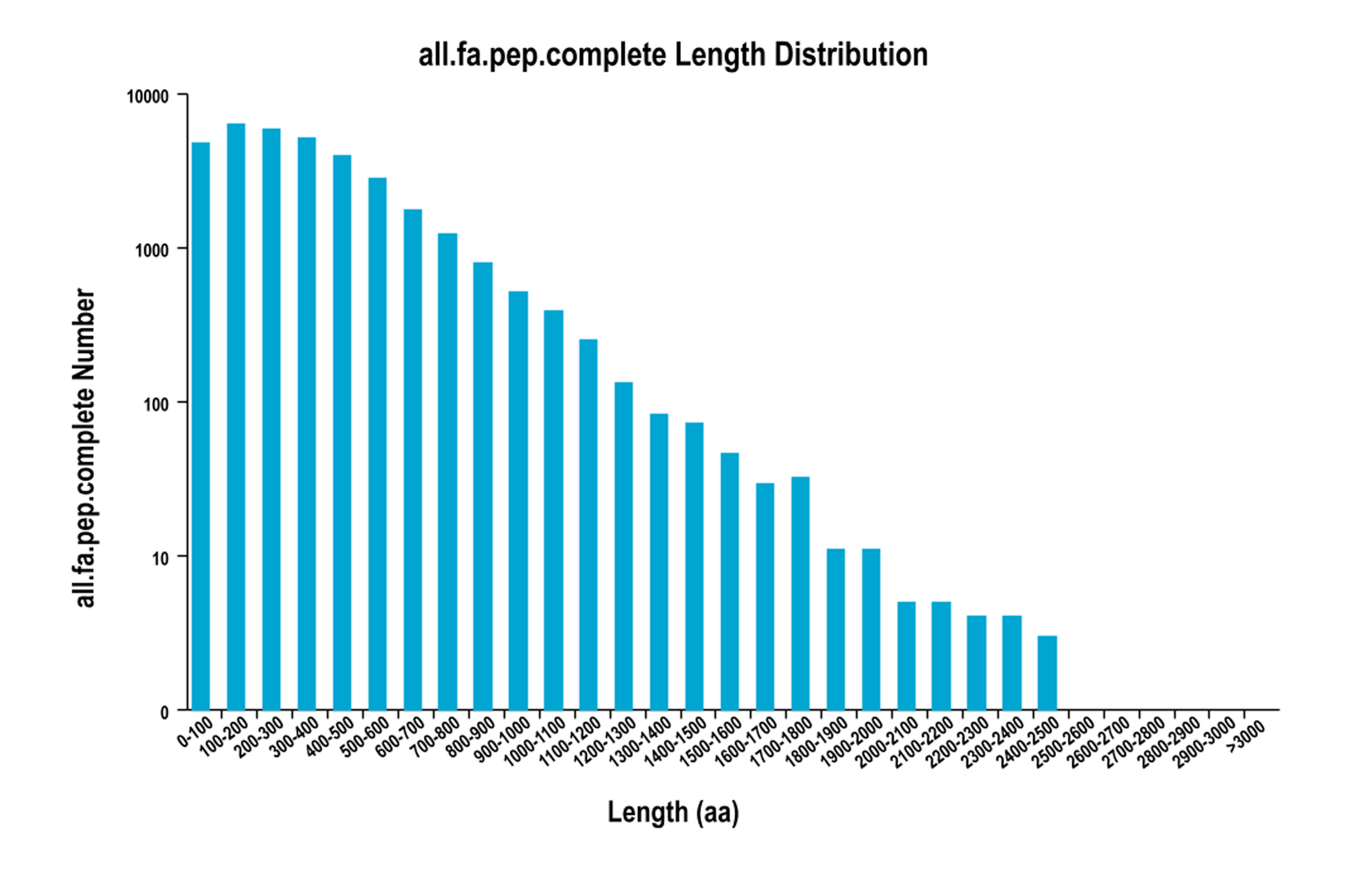

Supplement: Supplementary Figure 2 — The length distribution of the complete coding protein sequences in bone tissues of bighead carp. [file Image_2.JPEG]

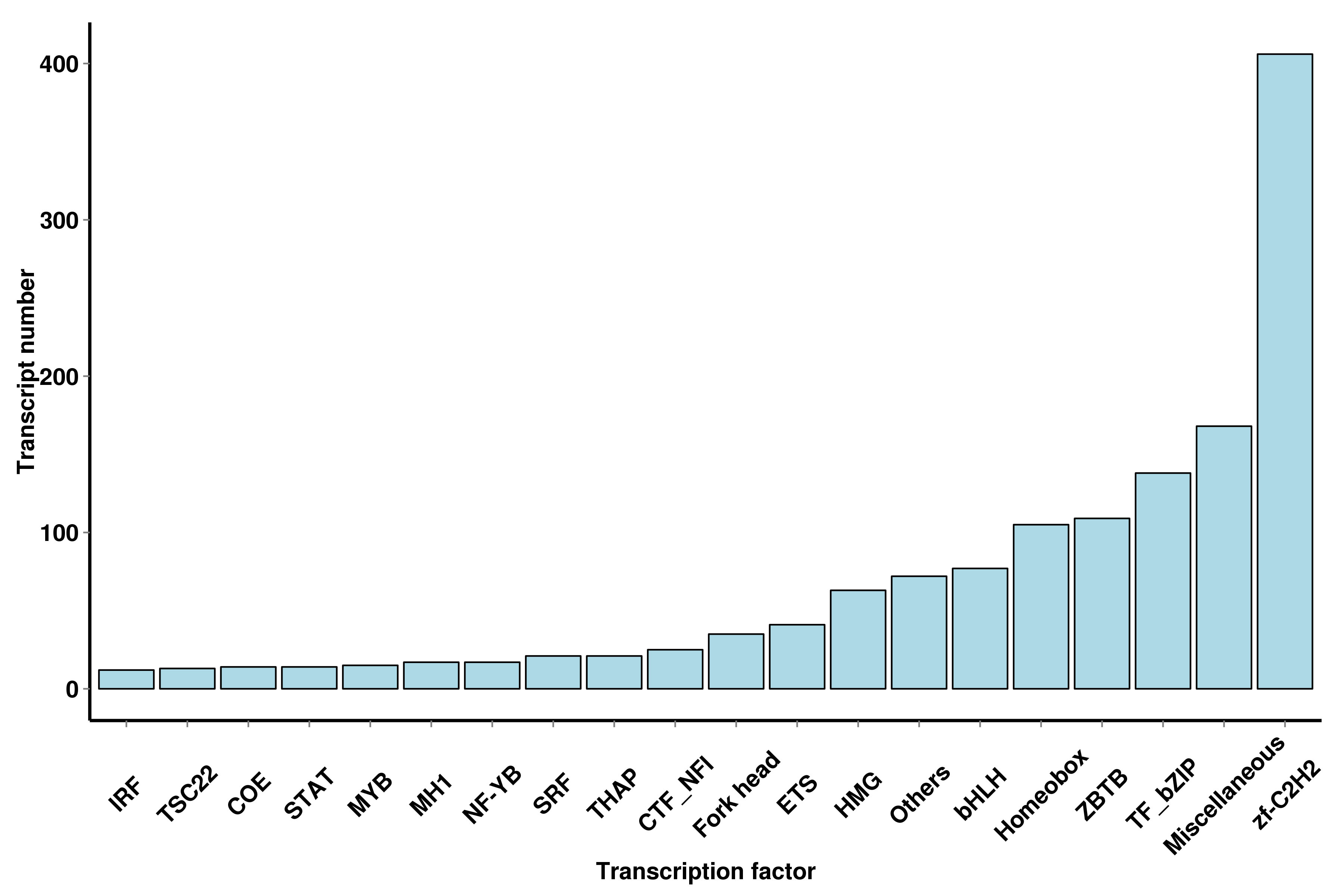

Supplement: Supplementary Figure 3 — Type distribution of transcription factors in bone tissues of bighead carp. [file Image_3.JPEG]
