# Supplementary material for: Identifying Candidate Genes Involved in the Regulation of Early Growth Using Full-Length Transcriptome and RNA-Seq Analyses of Frontal and Parietal Bones and Vertebral Bones in Bighead Carp (Hypophthalmichthys nobilis)
Source: Front Genet. 2021 Jan 15;11:603454. doi: 10.3389/fgene.2020.603454 (PMC7844397; doi:10.3389/fgene.2020.603454)
Supplement: Supplementary Table 1 — Summary statistics for RNA-seq information of the bighead carp frontal and parietal bone and vertebra transcriptomes. [file Table_1.DOCX]

**TABLE S1|** Summary statistics for RNA-Seq information of the bighead carp frontal and parietal bone and vertebra transcriptomes.

| **Samples** | **Read Number** | **Base Number** | **GC Content** | **%≥Q30** |
| --- | --- | --- | --- | --- |
| BF1 | 27,617,848 | 8,271,190,612 | 47.27 | 94.26 |
| BF2 | 22,121,535 | 6,621,556,546 | 50.64 | 94.78 |
| BF3 | 30,929,246 | 9,266,754,710 | 49.20 | 94.61 |
| BV1 | 26,882,927 | 8,055,582,444 | 49.27 | 93.86 |
| BV2 | 28,716,585 | 8,601,947,530 | 48.87 | 94.07 |
| BV3 | 23,182,318 | 6,943,950,542 | 48.79 | 93.93 |
| SF1 | 20,253,622 | 6,067,144,244 | 47.48 | 94.46 |
| SF2 | 20,925,384 | 6,265,282,148 | 49.36 | 94.91 |
| SF3 | 20,068,592 | 6,001,497,824 | 50.10 | 94.93 |
| SV1 | 25,882,124 | 7,752,013,154 | 47.22 | 93.16 |
| SV2 | 29,237,583 | 8,744,845,714 | 47.45 | 93.68 |
| SV3 | 31,544,161 | 9,446,473,284 | 50.31 | 93.03 |

Note: BF and BV indicate the abbreviation of the frontal and parietal bone and vertebra from the big group in bighead carp, respectively; SF and SV mean the abbreviation of the frontal and parietal bone and vertebra from the small group in bighead carp, respectively.

GC%: The percentage of proportion of guanidine and cytosine nucleotides among total nucleotides.

Q30: The percentage of bases with quality value larger than 30.
