# Supplementary material for: Identifying Candidate Genes Involved in the Regulation of Early Growth Using Full-Length Transcriptome and RNA-Seq Analyses of Frontal and Parietal Bones and Vertebral Bones in Bighead Carp (Hypophthalmichthys nobilis)
Source: Front Genet. 2021 Jan 15;11:603454. doi: 10.3389/fgene.2020.603454 (PMC7844397; doi:10.3389/fgene.2020.603454)
Supplement: Supplementary Table 7 — Primer sequences of the DEGs for qRT-PCR in bone tissues of bighead carp. [file Table_7.DOCX]

**TABLE S7|** Primer sequences of the DEGs for qRT-PCR in bone tissues of bighead carp.

| Gene | Forward primer (5'-3') | Reverse primer (5'-3') | Length  (bp) |
| --- | --- | --- | --- |
| *sugp1* | AGAAAGCCATCAGGAACCAC | CCTTTGCCCATCTCAGTCA | 145 |
| *atp1b* | TTTCATAACATACGGGACCTACA | GCTTGGAAAGGACCATTGTAT | 293 |
| *ptprc* | TTAGACGCATAAGAACACGACAT | TAAGACCAAGCATAGGACCAAAA | 112 |
| *ptp4a* | CGTTTGTCAGTGTGAGTGGAGA | TAAGACGGGCAGTCAGGTAGTA | 110 |
| *scd* | TAGAGGCTGCGTGACAATGA | GTTCCAGGTGTATTTAGAAGACGA | 162 |
| *plekhj1* | CTCCTCTGCCTTCTCCTCCC | AATCAAGCAAAAACCCCCAA | 102 |
| *afap1l2* | CCCAATCCCTACCCCTAAAG | TGGAGAGAACTTGAGCCTATCG | 98 |
| *msg4l* | AACCCCACTGGTCTGTATTTG | GTCGTGAAGCATTTGTAACCC | 155 |
| *ncoa5* | GAGAGATGAGGGGGGATAGC | CGTCTCCTCTTTCTTCCGATAGT | 185 |
| *glg1* | TGTGCGTGGTGCTGCTCTT | GCTATTTTTGTTAGTGAATGGGTATGT | 156 |
| *gpr126* | GAGCACACGCAACACCATCC | AACTCCATAACCAGCGGCAA | 250 |
| *sgk1* | TTTGACCCCGAGTTTACCG | GGGATGGCGTTTCTGCG | 169 |
| *β-actin* | TATCCTATTGAGCACGGTATTG | CCTGTTGGCTTTGGGATTC | 144 |

Gene abbreviations: *SURP and G-patch domain-containing protein 1* (*sugp1*), *sodium/potassium-transporting ATPase subunit beta* (*atp1b*), *receptor-type tyrosine-protein phosphatase C* (*ptprc*), *protein tyrosine phosphatase type IVA* (*ptp4a*), *stearoyl-CoA desaturase* (*scd*), *pleckstrin homology domain-containing family J member 1* (*plekhj1*), *actin filament-associated protein 1-like 2* (*afap1l2*), *microfibril-associated glycoprotein 4-like* (*msg4l*), *nuclear receptor coactivator 5* (*ncoa5*)*, golgi apparatus protein 1 (**glg1), G-protein coupled receptor 126(**gpr126), serine/threonine-protein kinase Sgk1(**sgk1).*
